# Editorial: Post-transcriptional regulation and its misregulation: From molecular basis to translational medicine

**DOI:** 10.3389/fcell.2022.1101576

**Published:** 2022-11-28

**Authors:** Ye Tian, Ai-Ming Yu, Chong Yin, Airong Qian

**Affiliations:** ^1^ Lab for Bone Metabolism, Xi’an Key Laboratory of Special Medicine and Health Engineering, Key Lab for Space Biosciences and Biotechnology, NPU-UAB Joint Laboratory for Bone Metabolism, Research Center for Special Medicine and Health Systems Engineering, School of Life Sciences, Northwestern Polytechnical University, Xi’an, Shaanxi, China; ^2^ Department of Biochemistry and Molecular Medicine, UC Davis School of Medicine, Sacramento, CA, United States; ^3^ Department of Clinical Laboratory, Academician (expert) Workstation, Lab of Epigenetics and RNA Therapy, Affiliated Hospital of North Sichuan Medical College, Nanchong, Sichuan, China

**Keywords:** post-transcriptional regulation, non-coding RNA, lncRNA, microRNA, tsRNA, diseases

The “central dogma”, which was proposed six decades ago, asserts that genetic information flows from DNA to protein through RNA. The following decades of research has featured RNAs mainly act as intermediates of protein translation, e.g., principally as transient copies of information for synthesizing genetically encoded proteins (mRNAs, messenger RNAs), components of ribosome (rRNAs, ribosomal RNAs), or adapters of translational machinery (tRNAs, transfer RNAs which read and transfer coding information from mRNA to ribosome *via* codons). With the progress of studying the “RNA world,” it is clear that the “end products” encoded in transcriptional units of the genome are not limited to proteins but comprised of a large variety of unique RNAs with diverse functions. Benefiting by the advances in RNA sequencing technologies, a multitude of non-coding RNA (ncRNA, RNAs lacking protein-coding regions) species have been identified, among them some are highly conserved, such as microRNAs (miRNAs), and others are generally non-conserved across species such as long ncRNAs (lncRNAs). Indeed, complex series of events constitute the cell’s gene expression programs which are controlled by transcriptional, post-transcriptional, and post-translational regulatory factors.

Post-transcriptional regulation plays important roles in gene expression at the RNA level, i.e., regulate mRNA stability or translational efficiency *via* ribosomes, and thus influences the functional outputs of genes (i.e., proteome content). Post-transcriptional gene regulation is governed primarily by different ncRNAs through various mechanisms. Recent studies have demonstrated post-transcriptional regulation as a critical mechanism underlying manifold eukaryotic biological processes. Therefore, defects in post-transcriptional gene regulation may lead to many types of diseases. Understanding the functions of ncRNAs in post-transcriptional gene regulation and its significance in normal tissue development and disease susceptibility is fundamental to improving the understanding of biology and developing new therapies. In this collection, 4 articles are published on the roles and functions of post-transcriptional gene regulation in various diseases.

Wu et al. scrutinized the lncRNAs being reported to affect cholangiocarcinoma (CCA) cell proliferation, migration, apoptosis, and drug resistance through numerous signaling pathways, supporting that some lncRNAs are potential therapeutic targets of CCA. They also pointed out that lncRNAs abnormally expressed in CCA could be detected in specific tissues or fractions, such as serum, bile, and exosomes, which harbored the potential as biomarkers for diagnosis or prognosis. The role of tRNAs has been expanded recently as tRNAs are a source of a group of small non-coding RNAs, namely tRNA-derived small RNAs (tsRNAs). Chu et al. summarized the current knowledge of tsRNA biogenesis and classification, as well as their functions and target gene regulation mechanisms. The existing evidence supports the involvement of tsRNAs in many disease processes, such as breast cancer, ischemic stroke, and osteoporosis. These findings highlight possible clinical implications of tsRNAs, including diagnosis and therapy.

MiRNAs are a class of small ncRNAs that control many biological processes, including development, differentiation, and metabolism. So, precise spatiotemporal management of miRNA levels is important for maintenance of normal physiological function. Pandey et al. highlighted that expression of polycistronic miRNAs was affected by their stem-base and terminal loop sequences. In addition, the processing kinetics of adjacent miRNAs in the same cluster also have some effects. These findings should improve the understanding of regulation mechanisms behind miRNA biogenesis and help to decipher variable actions of miRNAs in different biological processes. Lu extended the scope of this topic by illustrating how ribosome-associated quality control (RQC) tightly controlled the mRNA translation. The importance of RQC in neurodegenerative diseases, cancer, and viral infections was also discussed.

Altogether, this Research Topic provides a glimpse of the “kingdom” of post-transcriptional regulation and extends the knowledge on some aspects in the fields. While it does not cover all the topics announced in the open call for submissions, the present Research Topic is conducive to improve our understanding of ncRNAs in post-transcriptional regulation and provide insights into developing new treatments for relevant diseases.

